# Toward standardized MEP recording? Exploring the role of electrode configuration in TMS studies

**DOI:** 10.3389/fnhum.2024.1488438

**Published:** 2024-11-12

**Authors:** Ana Carolina Borges Valente, Lucas dos Santos Betioli, Lidiane Aparecida Fernandes, Daniela Morales, Lilian Pinto da Silva, Marco Antonio Cavalcanti Garcia

**Affiliations:** ^1^Programa de Pós-Graduação em Ciências da Reabilitação e Desempenho Físico Funcional, Faculdade de Fisioterapia, Universidade Federal de Juiz de Fora, Juiz de Fora, Minas Gerais, Brazil; ^2^Departamento de Física, Faculdade de Filosofia, Ciências e Letras de Ribeirão Preto, Ribeirão Preto, São Paulo, Brazil; ^3^Departamento de Educação Física, Universidade Federal de Ouro Preto, Ouro Preto, Minas Gerais, Brazil; ^4^Hospital Universitário – Unidade Santa Catarina, Universidade Federal de Juiz de Fora, Juiz de Fora, Minas Gerais, Brazil; ^5^Departamento de Biofísica e Fisiologia, Instituto de Ciências Biológicas, Universidade Federal de Juiz de Fora, Juiz de Fora, Minas Gerais, Brazil

**Keywords:** motor evoked potential, transcranial magnetic stimulation, corticospinal excitability, electromyography, surface electrodes placement

## 1 Introduction

Transcranial magnetic stimulation (TMS) has been widely used in investigating motor control under health and pathological conditions, bringing valuable insights into the neurophysiological underlying mechanisms mainly from the motor evoked potential (MEP) properties (Garcia et al., 2017; Moraes et al., 2023; Spampinato et al., 2023; Garcia et al., 2024). The MEP is an electromyogram (EMG) response resulting from a single TMS pulse recorded at rest or during submaximal voluntary contraction. Hence, the MEP helps us to interpret some properties of bulbar or corticospinal excitability (Spampinato et al., 2023) and, consequently, to assess different aspects of the motor system. Besides being adopted as a diagnostic parameter, the MEP is also a *key* reference in determining stimulation intensity in repetitive TMS (rTMS) treatment protocols (Turi et al., 2021, 2022). Consequently, substantial methodological progress has been made to enhance the consistency of TMS recording. In this context, ensuring the correct positioning of the TMS coil on the patient's head to evoke reliable muscle responses and reduce variability in MEP properties, such as its peak-to-peak value (MEP_P − P_), represents a *sine qua non*-condition. This requirement has become even more prominent with the development of neuronavigation systems (Krings et al., 2001; Ruohonen and Karhu, 2010) and their ongoing refinements (Souza et al., 2018a; Matsuda et al., 2023). Moreover, the introduction of autonomous robotic handling (Kantelhardt et al., 2010; Harquel et al., 2016) brought additional significant advantages for more effective control of the TMS coil, which, since their advent, also become progressively accurate for this purpose (Matsuda et al., 2024). However, while we observe many advancements ensuring precision in TMS applications, we can also note, for instance, a rather expressive number of studies using different surface electrode montages (Moraes et al., 2023; Koponen et al., 2024) to record the EMG signal. Thus, depending on the montage and dimension of the surface electrodes, it will be possible to identify significant differences in specific MEP properties, such as spectral composition, number of phases, and MEP_P − P_.

Nevertheless, it is worth emphasizing that although there are some recommendations for electrode montages for registering the surface EMG (sEMG) in many aspects of human performance (Hermens et al., 2000), there seems to be no agreement regarding MEP recording. Indeed, Garcia et al. (2017, 2020, 2023) have addressed this issue, highlighting how the lack of standardization could lead to misinterpretations of the bulbar- and corticospinal excitability profile.

Therefore, the present manuscript discusses how different surface electrode montages contribute to MEP properties and their consequences in interpreting motor cortical excitability. In addition, we present findings derived from a pilot trial in which the myoelectric activity of *biceps brachii* (BB) was recorded using multichannel electromyography (HD-sEMG) to shed light on the issues presented therein.

## 2 The surface electromyography in muscle activity assessment

Surface EMG is a widely used technique for the non-invasive assessment of muscle activity (Temesi et al., 2014; Peres et al., 2018). However, similarly to other biological signals such as the electrocardiogram and electroencephalogram, the location of surface electrodes is also crucial in ensuring the reliability and accuracy of the recorded sEMG signal (Garcia and Vieira, 2011; Merlo et al., 2021). In light of the many variables to be aware of in the sEMG signal acquisition process, some initiatives have introduced recommendations to optimize this signal recording. For instance, the SENIAM project (Surface ElectroMyography for the Non-Invasive Assessment of Muscles; www.seniam.org; Hermens et al., 2000) and, more recently, the CEDE project (Consensus for Experimental Design in Electromyography; https://isek.org/cede-project/; Besomi et al., 2019) represent two initiatives focused on ensuring the widespread establishment of guidelines on the use of sEMG. It is worth noting that although the SENIAM recommendations were not explicitly designed for MEP recording, they have also been widely used in TMS studies (Mrachacz-Kersting et al., 2021; Kindred et al., 2021; Rodriguez et al., 2022; Koponen et al., 2024).

In turn, the International Federation of Clinical Neurophysiology (IFCN; https://www.ifcn.info/) (Groppa et al., 2012) has broadened the debate on recording myoelectric activity, whether invasive or not, which includes MEP recording. It is interesting to highlight that SENIAM/CEDE and IFCN recommend different surface electrode montages to record the sEMG signal. However, how can both surface electrode montages affect the recording and, consequently, the MEP interpretation?

## 3 SENIAM/CEDE vs. IFCN recommendations for surface electrode montages and their influence on MEP properties

Regarding surface electrode positioning, the SENIAM/CEDE project recommends placing two electrodes (1 cm diameter; ~100 mm^2^) in a bipolar configuration between the innervation zone (IZ) and the muscle-tendon junction. It provides detailed instructions on the optimal placement of electrodes for different muscle groups, considering anatomical landmarks and muscle fiber orientation. Depending on the muscle size, an interelectrode distance of 1 to 2 cm is recommended, which allows for the adjustment of the detection volume. Aligned with these recommendations, the MEP is given by a muscle region circumscribed by the myoelectric activity of the portion covered by the two surface electrodes. As a result, the MEP_P − P_ should be positively correlated with the interelectrode distance.

Nevertheless, this registration model has two critical concerns: a greater interelectrode distance will lead to a higher probability of detecting MEPs from adjacent muscles (*crosstalk*; van Elswijk et al., 2008), and an increase in the contribution of lower frequencies to the MEP content. In both cases, the interpretation of cortical excitability may be jeopardized. Moreover, since the spatial distribution of myoelectric activity along the muscle is not necessarily homogeneous, as has been observed from multichannel or high-density electromyography (HD-sEMG) (van Elswijk et al., 2008; Souza et al., 2018b), the SENIAM recommendations may not necessarily provide significant advantages for MEP recording.

On the other hand, the IFCN suggests using a different surface electrode placement based on a belly-tendon montage (*pseudomonopolar*) for MEP recording. Based on Groppa et al. (2012), the active electrode (E_A_) is placed on the muscle belly that, according to Stålberg et al. (2019), corresponds to the IZ, and the other (E_O_) over the tendon or an inactive location. Nevertheless, assuming a correlation between the muscle belly and the IZ is improper (Barbero et al., 2012). Despite the limitations mentioned, its relevance is widely recognized in basic and clinical neurophysiology (Ah Sen et al., 2017; Nikolov et al., 2021).

Although TMS users widely adopt the SENIAM and IFCN recommendations, Garcia et al. (2017) reinforced the lack of agreement regarding their surface electrode montages, which can lead to misinterpretation of MEP properties. Moreover, the authors hypothesized that conventional electrode placement protocols, such as those recommended by SENIAM and IFCN, might not fully account for the inhomogeneous nature of muscle architecture and motor unit action potential (MUAP) propagation resulting from the TMS pulse. The central hypothesis is that a suitable surface electrode montage should provide a more reliable measure of the excitability of the bulbar- or corticospinal pathway, i.e., greater MEP_P − P_ and lower coefficient of variation. Consequently, the authors suggest adopting a pseudomopolar montage, as indicated by IFCN, but by ensuring the correct IZ localization, such as by using HD-sEMG. The rationale for placing E_A_ at the IZ is based on the higher likelihood of MUAPs coherent summation, which results in a maximum MEP_P − P_ even at a low TMS pulse intensity.

To test the eventual differences in MEP recording from SENIAM and IFCN recommendations, Garcia et al. (2020) investigated the impact of their protocols on the MEP_P − P_ (***a***. SENIAM protocol; and ***b***. E_A_ [over IZ] and E_O_ [over bone prominence]; reference electrode over bone prominence C7) from *biceps brachii* (BB), *flexor carpi radialis*, and *flexor pollicis brevis* muscles. The IZ-bone prominence montage resulted in ~3.5 to 6.1 times higher MEP_P − P_ than SENIAM recommendations at the same TMS pulse intensity. Subsequently, Garcia et al. (2023) also compared three electrode montages on the MEP_P − P_ (***a***. IZ-bone prominence; ***b***. IFCN [muscle belly-bone prominence]; and ***c***. E_A1_-E_A2_ [bipolar over muscle belly] with a wide interelectrode distance) from BB muscle. MEP_P − P_ were ~2.0 to 2.6 greater for the IFCN protocol than the other two surface electrode montages. The IZ-bone prominence montage provided MEP_P − P_ ~1.3 to 1.6 greater than montage ***c*** (E_A1_-E_A2_ [bipolar over muscle belly]). Although these findings contradict the hypothesis postulated by Garcia et al. (2017), the authors suggest that this potential difference could be explained by muscle characteristics such as size, muscle fiber organization, and IZ distribution, which should also serve as a basis for guiding the formulation of the most appropriate electrode montage for MEP recording.

## 4 The impact of electrode placement variability on MEP recording and clinical decision-making

Since electrode placement is a determinant in accurately acquiring the sEMG signal, we may conjecture that it directly affects MEP properties. Consequently, variability in surface electrode montages across studies and clinical settings can lead to significant discrepancies in data interpretation. Such inconsistency can produce conflicting data on bulbar- or corticospinal excitability evaluation. One of the primary consequences of divergent surface electrode placements is the risk of overestimating or underestimating the bulbar- or corticospinal excitability. For example, based on the reports presented above (Garcia et al., 2020, 2023), divergences in MEP_P − P_ are particularly concerning in pathological conditions where accurate assessment is critical for diagnostic and rehabilitative purposes, as in rTMS protocols. Therefore, comparing outcomes across different studies or within longitudinal patient assessments from different surface electrode montages becomes challenging. Moreover, it may obscure the true extent of motor impairment or mask the potential benefits of a therapeutic intervention, leading to suboptimal clinical decision-making.

## 5 The potential of high-density sEMG in enhancing MEP recording protocols

**Figure 1:**
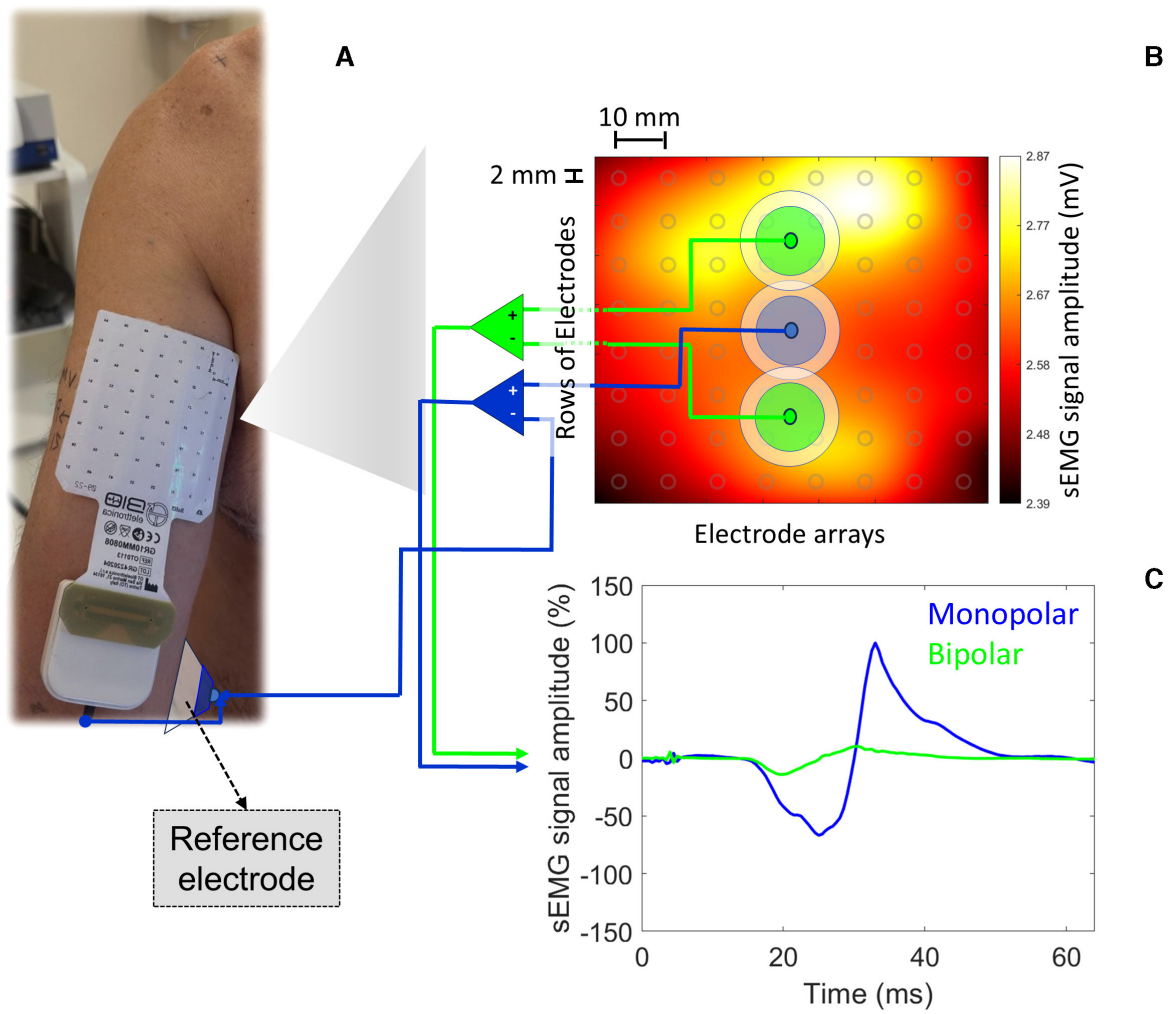
MEP_P − P_ were obtained from two simulated surface electrode montages (bipolar vs. pseudomonopolar) based on real myoelectric activity recorded from HD-sEMG. In **(A)**, the square matrix (8 × 8; SESSANTAQUATRO, OT Bioelettronica, Italy) of surface electrodes (electrode diameter: 2 mm; inter-electrode distance: 10 mm) is centered on the BB muscle belly (rows and columns 4 and 5). A conventional surface electrode (E_O_ [reference electrode]; 1 cm in diameter; Ag/AgCl) was placed on the medial epicondyle of the humerus. In **(B)**, we identify the average map of myoelectric activity (mV) derived from twenty single TMS pulses at 120% of the resting motor threshold performed using a parabolic coil (model: MMC-140-II) and the R20 system (Magventure, Denmark). Locations defined by two different simulated surface electrode configurations (bipolar [interelectrode distance: 4 cm] vs. pseudomonopolar; electrode detection area: 100 mm^2^) are also highlighted, from which the MEPs presented in **c** were extracted. A single simulated E_A_ (electrode detection area: 100 mm^2^) adopted for the pseudomopolar montage is presented in the muscle belly, from where the blue MEP was extracted. Based on the myoelectric activity generated by a single TMS pulse, it is possible to observe the lack of homogeneity in the myoelectric activity profile under the matrix area from a monopolar registration. In **(C)**, arbitrarily normalized MEPs from the positive peak of MEP originated from the pseudomonopolar montage. The MEPs derived from the bipolar and monopolar surface electrode montages are in green and blue, respectively. The bipolar montage provided a MEP_P − P_ ~85% lower than that obtained from the pseudomonopolar. The volunteer was a 43-year-old right-handed male with no neurological or musculoskeletal disorders.

The advent of HD-sEMG represents a significant leap forward in recording myoelectric activity from high spatial resolution. HD-sEMG offers a broader perspective on muscle activity by recording from multiple sites within the same muscle. Thus, it can help understand the complex spatial dynamics of motor unit recruitment during TMS-induced motor responses. For instance, Figure 1 illustrates the myoelectric activation recorded from the BB muscle (matrix dimension: 8 × 8 electrodes; electrode dimension: 2 mm diameter; interelectrode distance: 1 cm) by applying a single TMS pulse at 120% of the resting motor threshold and over its *hotspot* (left primary cortex [M1]). Under resting conditions, at least 20 single TMS pulses were applied at BB *hotspot*. The bipolar montage provided a MEP_P − P_ ~85% lower than that obtained from the pseudomonopolar. Since the muscle activation is not uniform along the whole muscle, depending on the local over the muscle chosen and the simulated surface electrode montage adopted (bipolar vs. pseudomonopolar), one can obtain different MEP_P − P_ values. Therefore, HD-sEMG can help identify how different muscle areas contribute to the MEP profile, revealing information about the heterogeneity of motor unit activation.

Consequently, it also allows for detecting subtle changes in muscle activation patterns that may be missed by unsuitable surface electrode montages for MEP recording. A high-resolution mapping can help isolate specific regions of muscle activation most responsive to a single TMS pulse, leading to more targeted and effective stimulation protocols. Thus, using HD-sEMG with TMS could pave the way for more assertive electrode placement protocols. As a result, by optimizing electrode positioning based on individual muscle activation patterns, researchers and clinicians could improve the reliability of MEP recordings and gain deeper insights into motor control processes. Such protocols would enhance the accuracy of MEP recordings and ensure greater consistency across studies, leading to more reliable conclusions about motor performance and neuromodulatory outcomes.

## 6 Conclusion

The inconsistency across studies underscores the need for consensus on standardized protocols for MEP recording. These divergences affect motor performance interpretation and hinder the development of universal benchmarks for neuromodulation treatments like rTMS. As rTMS use grows in clinical and research settings, a better understanding of how electrode placement impacts MEP properties will refine its role in rehabilitation and motor performance.

Integrating HD-sEMG into TMS protocols offers the potential for improving MEP recording accuracy. Enhancing spatial resolution can reduce risks from variable electrode placements, enabling more precise motor function assessments in healthy and pathological populations. As these technologies evolve, they will likely shape future neurophysiological research and clinical practice, supporting the creation of standardized electrode placement protocols.
